# Audio Spatial Representation Around the Body

**DOI:** 10.3389/fpsyg.2017.01932

**Published:** 2017-11-03

**Authors:** Elena Aggius-Vella, Claudio Campus, Sara Finocchietti, Monica Gori

**Affiliations:** Unit for Visually Impaired People, Center for Human Technologies, Istituto Italiano di Tecnologia, Genoa, Italy

**Keywords:** spatial representation, auditory perception, sensory interaction, blindness, spatial cognition

## Abstract

Studies have found that portions of space around our body are differently coded by our brain. Numerous works have investigated visual and auditory spatial representation, focusing mostly on the spatial representation of stimuli presented at head level, especially in the frontal space. Only few studies have investigated spatial representation around the entire body and its relationship with motor activity. Moreover, it is still not clear whether the space surrounding us is represented as a unitary dimension or whether it is split up into different portions, differently shaped by our senses and motor activity. To clarify these points, we investigated audio localization of dynamic and static sounds at different body levels. In order to understand the role of a motor action in auditory space representation, we asked subjects to localize sounds by pointing with the hand or the foot, or by giving a verbal answer. We found that the audio sound localization was different depending on the body part considered. Moreover, a different pattern of response was observed when subjects were asked to make actions with respect to the verbal responses. These results suggest that the audio space around our body is split in various spatial portions, which are perceived differently: front, back, around chest, and around foot, suggesting that these four areas could be differently modulated by our senses and our actions.

## Introduction

Audio spatial representation is crucial for everyday interaction with the environment. Acoustic studies have shown that the ability to localize sounds in space depends on anatomical and physiological properties of the auditory system as well as on behavioral cues. These cues are based on different binaural mechanisms, like the difference in time and the difference in sound intensity of the audio signal processed by the two hears (Letowski and Letowski, 2012). The time difference (ITD) relies on difference in the time it takes a sound to reach the closer ear and the farther ear. This is the dominant binaural cue for low frequency sound source localization. The Interaural level-difference (ILD) is the difference in the level of sound reaching the ear closer to the sound source and that reaching the farther, shadowed, ear. This is an important cue for high frequency sound (Macpherson and Middlebrooks, 2002; Middlebrooks, 2015). It is difficult, for the human brain, to disambiguate the position of the sound placed in front of or behind the body. This spatial perceptual ambiguity is known as the cone of confusion (Wallach, 1938), an imaginary cone extending outward from each ear, representing sound source locations producing the same interaural differences. When the binaural information correlates equally well with two opposite spatial locations, it is possible to incur in reversal errors (Carlile et al., 1997; Scharine and Letowski, 2005). In this specific condition, the estimation of the sound source location is reported in the opposite direction to the actual sound source location. Despite their significant role in horizontal localization, binaural cues are less efficient for vertical localization or front–back differentiation. Front–back (FB) and back–front (BF) errors are the most common reversal errors. However, they are rare for open ear conditions and are most frequent for sound sources located on or near to the median plane (Makous and Middlebrooks, 1990). Usually, front–back errors dominate back–front errors, but their proportion depends on various factors, such as the visibility of the sound sources (Chasin and Chong, 1999). Monaural cues are more powerful in differentiating between specific positions on the surface of the cone of confusion, as they do not depend on the presence of two ears. They result mostly from sound absorption by the head and the outer ear (pinna) (Steinhauser, 1879; Batteau, 1967; Gardner and Gardner, 1973; Musicant and Butler, 1984; Lopez-Poveda and Meddis, 1996). Several studies have reported that localization error of static sounds is more accurate in the frontal space, at head level, while error increased in the regions behind the head (Oldfield and Parker, 1986).

From a cognitive point of view, the space around us is split in several regions based on anatomical and neural activities. Electrophysiological studies (di Pellegrino and Làdavas, 2015), studies on neglect patients (Vallar et al., 1995; Farnè and Làdavas, 2002; Saj and Vuilleumier, 2007; Viaud-Delmon et al., 2007), and studies on the peripersonal space (Rizzolatti et al., 1997; Aimola et al., 2012; Cléry et al., 2015; Serino et al., 2015) show that our brain does not represent space as a unitary dimension. Evidence also suggests that space representation is split up into different portions in relation to the body position, i.e., near and far space (Ladavas and Serino, 2008), frontal and rear space (Saj and Vuilleumier, 2007; Viaud-Delmon et al., 2007; Zampini et al., 2007; Occelli et al., 2011), space around specific parts of the body (di Pellegrino and Làdavas, 2015; Serino et al., 2015), and space above and below the head in the frontal field (Finocchietti et al., 2015). Studies on neglect patients (De Renzi et al., 1989; Brozzoli et al., 2006; Jacobs et al., 2012) and on healthy people (Godfroy-Cooper et al., 2015) show that spatial representation can be affected by a specific sensory modality (i.e., vision) and, at the same time, can be intact for other sensory channels (i.e., touch and hearing). Interestingly, studies on agnosia (Coslett, 2011) have shown that object representation could also be selectively impaired with one sense and yet be intact with the others. These results suggest that different body regions can be differently represented by different sensory modalities in our brain.

Body movements also have an important role in spatial cognition. This idea is supported by the motor-oriented approach, which assumes that spatial relationships are coded by body movement in the space (Paillard, 1991). Our brain represents space based on the possibility to directly act on it (within/outside hand-reaching distance). Moreover, our actions can change the representation of space, in peripersonal space for example, the training with a tool modifies the extension of the body space, that in turn affects spatial representation, making what was previously far away seem closer (Berti and Frassinetti, 2000). With regard to the auditory spatial representation, Hofman et al. (1998) found that the human auditory system is able to adapt in response to altered spectral cues, which do not interfere with the neural representation of the original cues. This suggests that the hearing system is highly plastic in interpreting new acoustical cues. Finally, as motor and auditory system are strictly related in the brain, neuroimaging studies have shown that simply listening to an auditory rhythm engages motor areas in the brain (Grahn and Brett, 2007).

All these findings together suggest that different parts of the space around us may be differently organized, based on the dominant perceptual system for that portion of space and they could be differently shaped by motor activity. To date, most studies have focused on studying audio perception in the front and back space (Farnè and Làdavas, 2002; Zampini et al., 2007) or in high and low space (Heed and Röder, 2010; Scandola et al., 2016) separately, or they have studied the effect of actions on audio perception (Weiss et al., 2011; Timm et al., 2014; Viaud-Delmon and Warusfel, 2014). Here we study all these aspects in a unique framework by using unitary approach. With this goal in mind, subjects were requested to perform an audio perception task in the frontal and back zone, at high and low level, and giving a motor or verbal response. Firstly, we investigated how front and back auditory spaces are perceived; to do this, we manipulated sound location by delivering stimuli in the frontal and rear space. To investigate whether frontal and rear auditory space differ for upper and lower body portions, we manipulated sound elevation, by delivering stimuli around the chest area and around the foot area. To investigate the influence of action on audio perception, subjects had to report the sound position with a body movement, in one condition, or by giving a verbal answer in another condition. Finally, we investigated whether sound features could influence the localization of auditory stimuli by presenting both dynamic and static sounds. Results suggest that auditory perception is different for different body portions and modulated by actions. These findings suggest that senses and actions have a different weight in representing/shaping spatial representation of auditory stimuli delivered around the body.

## Materials and Methods

### Subjects

Twenty-six healthy participants took part in the study (13 females: average age 25 ± 3 years and 13 males: average age 30 ± 12 years). All participants had a similar level of education (at least an Italian high school diploma, indicating 13 years of schooling). A group of 11 people (4 males: average age 27 years ± 4 and 7 females: average age 27 years ± 5) performed motor pointing tasks, while a group of 15 people (9 males: average age 30 years ± 14 and 6 females: average age 25 years ± 3) performed verbal pointing tasks. All participants confirmed they were right handed and right footed, and they had normal or corrected-to-normal visual acuity and no history of hearing impairment. All participants provided written informed consent in accordance with the Declaration of Helsinki. The study was approved by the ethics committee of the local health service (Comitato Etico, ASL3 Genovese, Italy).

### Set-Up and Protocol

**FIGURE 1:**
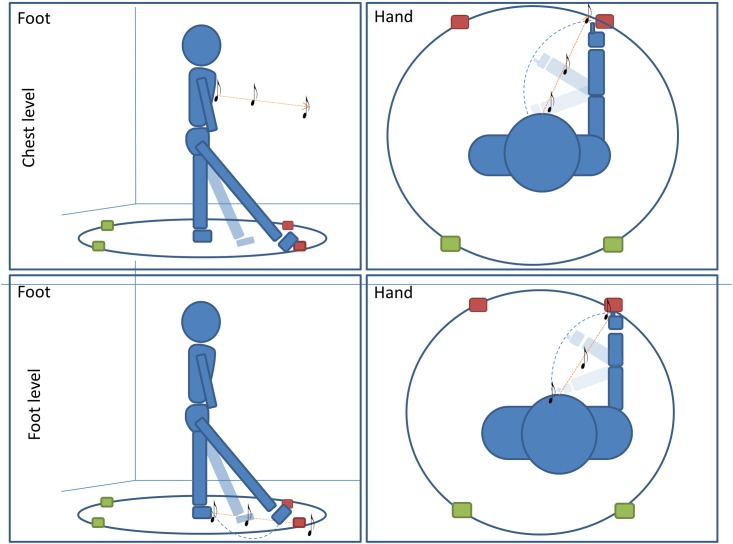
Pointing task: subjects performed four conditions of a pointing task. In two conditions subjects were asked to point with (1) foot (first column)or (2) hand (second column) to the final position of a sound moving radially from the subject to one of the four positions. In the other two conditions, subjects were asked to orally locate a (1) moving or (2) static sound delivered at one of the four positions on the circle (red for frontal point, green for back point). For all conditions, sounds were delivered at high (chest, first line) level and at low (foot, second line) level.

The experiment was performed in a dark room. The apparatus consisted of a circle (radius = 50 cm) drawn on the floor in the center of the room (far from each wall). Participants stood at the center of the circle and remained in this position for the entire duration of the experiment. Four different positions were marked on the perimeter of the circle; two positions were placed in the frontal portion of the space at -20 and 40° respectively, two positions were placed in the back portion of the space at 160° and 220° respectively (see **Figure 1**). All four positions were evaluated five times, for a total of 20 trials per condition (80 trials per participant).

All subjects enrolled were assigned to one of two groups and each group performed two conditions of a sound motion localization task (**Figure 1**). The first group of subjects had to (1) locate a dynamic sound with a motor pointing task using the foot and (2) locate a dynamic sound with motor pointing task using the hand. The second group of subjects had to: (1) locate a dynamic sound with a verbal response by a localization label, and (2) locate a static sound with a verbal response by a localization label. All conditions were divided into two randomized blocks in which the sound was delivered at high (chest) or low (foot) levels.

Sound stimuli were delivered by a digital metronome (Keuwlsoft, United Kingdom) set as single pulse (with no sub pulse), intermittent sound at 180 bpm; it showed a component at 1000 Hz. All subjects confirmed that they could hear the sound clearly. The same experimenter (EAV) administered all the tasks to all subjects; she was trained to keep the velocity of the moving sound constant, so that all features of stimulus were consistent across trials, positions, conditions and groups. She moved around the circle, holding the sound source, in order to produce the sound stimuli. We adopted a metronome as stimulus, as we were interested in understanding the interaction between motor and auditory systems in representing space, and the rhythmic sound was found to activate motor system (Grahn and Brett, 2007). All participants were blindfolded before entering the experimental room in order to avoid side effects related to setup or room observation.

During the conditions for which a motor response was required (first group), four spherical markers were placed on the subjects’ hands and feet for motion tracking: one on each distal phalange of the two index fingers and one on each distal phalange of the two big toes, (Vicon Motion Systems Ltd., United Kingdom). Four other markers were placed on the four positions on the circle (**Figure 1**); the markers placed on the floor represented the end point of the sound stimuli, i.e., where subjects should point. These markers were used to compute accuracy and precision. This paradigm was developed starting from the setup used in (Sara et al., 2017). All pointing movements were carried out on the same level of the effector used, regardless of the sound elevation (elevation did not change within condition); in order to avoid trunk torsions and to increase pointing accuracy, subjects were free to use the right or left effector. In this way, the two spaces taken into account maintained the same relationship in relation to the body space throughout all trials (i.e., avoiding torsions, the head was always aligned with the body, making frontal and back space constant in relation to body and head axes). It is important to note that, in the condition where pointing was performed with the hand, at starting position subjects were required to keep their hands on their chest (level at which the high sound was delivered). This arrangement was adopted because moving sounds within peripersonal space (PPS) modulate the motor system (Finisguerra et al., 2015). Participants were instructed not to move the effector until the end of the audio motion and to keep their head straight. Each time, after pointing, subjects returned to the original central position.

During the conditions for which a verbal response was requested (second group), subjects specified the sound source location by selecting it from a set of specifically labeled locations (“front-left, front-right, back-left, back-right”). When asked to localize the static sounds, the experimenter placed the sound in one of the four possible positions marked on the circle, while when asked to localize the dynamic sounds the experimenter moved the sound from the subject toward one of the four positions.

### Data Analysis

Kinematic data were post-processed and analyzed using Matlab (R2013a, The Math Works, United States), while R program (R Development Core Team, New Zealand) was adopted for the statistical analysis. Localization error and spatial precision (on *x* and *y*-axes) were computed for each participant and for each spatial position. The *x-* and *y*-coordinates in relation to subject position were obtained by a custom made program in Matlab. Localization error (also called error) was calculated as the distance (in cm) between the end-point position signaled by the participant and position of the reference marker placed on the circle. The error was averaged based on the number of trials per position and on the number of participants.

To better explore the meaning of the localization error, we calculated bias separately on *x*-and *y*-axes by subtracting the coordinates of the reference marker from the coordinates corresponding to the average end-point positions signaled by the participants.

The precision on *x*-and *y*-axes was calculated as standard deviation for each point, averaged among subjects. We supposed that points with the same longitudinal position in relation to the body were homogenous in localization error (as they share the same area as the body). A *t*-test confirmed our hypothesis, allowing us to group the four points into two spaces, i.e., front and back. In order to understand whether auditory space representation is influenced differently by sound elevation (around chest and around foot), effector used to point (hand, foot) and longitudinal position (front, and back space), we performed five repeated measure ANOVAs, independently considering localization error, bias, precision on *x*-axis and precision on *y*-axis. In the verbal tasks, subjects were required to indicate the end point of the sound by naming. We fitted a beta regression model for proportions of responses given by the subjects in each quadrant, therefore considering proportions as a function of sound level (high level vs. low level), longitudinal position (front vs. back space) and transversal position (right vs. left). We calculated Analysis of Deviance Tables (using Type II Wald chi-square tests) for the models using the Anova function of the car package (Fox and Weisberg, 2011). For significant effects, we performed *post hoc* comparisons using the lsmeans package (Lenth, 2016), which computes and contrasts least-squares means (predicted marginal means). We adopted MVT P adjustment, which uses a multivariate *t* distribution. Contrasts, with *P* < 0.05 were considered as significant (*P* corrected are reported). The same analysis was also adopted to investigate front–back error in the motor pointing task.

## Results

Three models were used to analyze our data. For data regarding the motor point, we adopted an Anova on localization error and anova on bias on *x-* and *y*-axes. Data from every tasks were analyzed with a beta regression on proportion of responses.

All model showed a particular salience of the back space, with difference between high and low space. While in the low space subjects localized frontal sound in the back, displaying a great number of front–back errors; in the high space, the front–back error was still present in the oral conditions, while in the motor condition, the error was better explained by a shift toward the back of sound perception.

### Localization Errors

**FIGURE 2:**
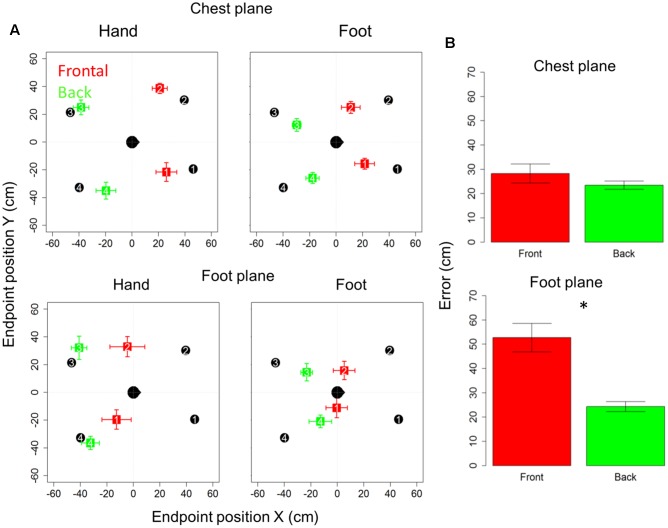
Bias and localization error in motor pointing task: **(A)** upper and lower rows represent sound levels: high and low, respectively; left and right columns represent the effector used: hand and foot, respectively. In each quadrant, the subject is indicated by the head at the center facing toward positive *x*-values. Black circles represent the four positions to be located, while colored squares denote the average of locations actually located: red and green squares, respectively, refer to the front and back longitudinal spaces, respectively. As can be seen, there is greater bias at foot level (red squares are shifted toward the back), while at chest level the bias disappears (green dots are almost superimposed on black dots). **(B)** Reports localization error (distance in cm between the end-point position signaled by the participant and position of the reference marker placed on the circumference). As can be seen, similar localization in front and back space is reported for sounds delivered at chest level, while greater localization error in the frontal space appears when sounds are delivered at foot level. Significant differences are illustrated (^∗^*P* < 0.05).

**Figure 2A** reports localization error and precision (standard error for each point averaged among subjects), in *x-* and *y*-axes for the motor audio pointing. Specifically, upper and lower rows represent sound levels, chest and foot, respectively, while left and right columns represent hand and foot effectors. In each quadrant, the subject is indicated by the head at the center (*x* = 0 cm, *y* = 0 cm) and he/she is facing toward positive *x*-values. Black circles represent the four targets to be located, while colored squares denote the average of locations actually located (indicated by numbers): red and green squares refer to the front and back longitudinal spaces, respectively.

**FIGURE 3:**
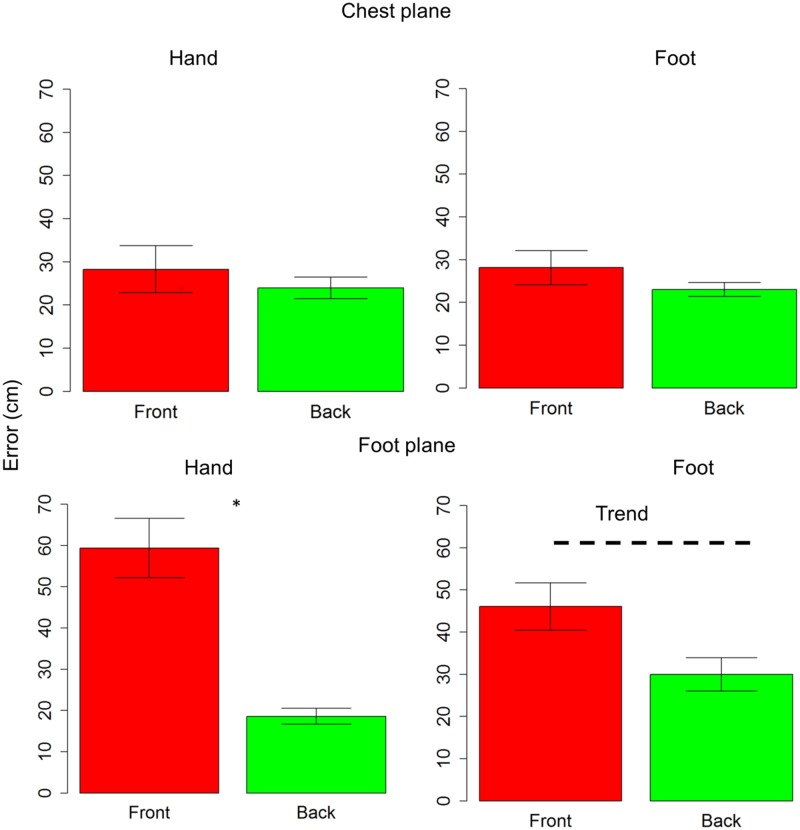
Effectors influence localization in frontal and back space at foot level: upper line shows localization error for sounds delivered at chest level, while lower line reports localization error for sounds delivered at foot level. The first column reports data for hand pointing, the second column represents pointing with the foot. The red bar refers to frontal space, while the green bar represents back space. As can be seen, at chest level subjects were similarly accurate with both effectors, in both spaces. At foot level, a significant difference between frontal and back space is reported when pointing with the hand. The same pattern (trend) is also reported when pointing with foot. Significant differences are illustrated (^∗^*P* < 0.05).

Subjects were generally more accurate (smaller localization error) in the back space than in the frontal space [*t*_(10)_ = 3.5, *P* = 0.006] and in the space around chest, than the space around foot [*t*_(10)_ = 5.4, *P* = 0.0003]. However, anova on localization errors showed that sound elevation (chest vs. foot level) significantly influences sound localization on the longitudinal plane (front vs. back) [*F*_(1,10)_ = 21, *P* = 0.001]. Indeed, when the sound was delivered at chest level, subjects showed similar error in localizing sounds coming from both the frontal and back space [*t*_(10)_ = 0.9, *P* = 0.7], while, when the sound was delivered at foot level, there was greater localization error in the frontal space than in the back space [*t*_(10)_ = 4.8, *P* = 0.001]. No significant localization errors were observed when the sound was presented at chest level (squares are almost superimposed on circles in the upper line in **Figure 2A**). Contrarily, at the foot level, frontal sounds were mostly perceived as coming from the back and a strong localization error emerged (lower line in **Figure 2A**). **Figure 2B** reported the average error, considering frontal and back regions. Interestingly, precision was equal for different sound elevations, on both: the *x*-axis [*F*_(1,10)_ = 1.8, *P* = 0.2] and the *y*-axis [*F*_(1,10)_ = 2.6, *P* = 0.1]. In order to test the role of the effectors (hand or foot) on the audio spatial bias, we carried out the task twice, asking to the subjects to point with either the hand or foot. The localization error on the longitudinal plane (front vs. back) was influenced by the effector used and sound level [*F*_(1,10)_ = 8.3, *P* = 0.02]. **Figure 3** compares error in localizing frontal (red bar) and back sound (green bar), when pointing with the hand (left column) and with the foot (right column), at both sound elevations, chest level (upper line) and foot level (lower line). As can be seen, with sounds delivered at chest level, subjects were similarly accurate in localizing frontal and rear sounds with both effectors, hand [*t*_(10)_ = 0.6, *P* = 1] and foot [*t*_(10)_ = 1.2, *P* = 1]. At foot level, subjects displayed higher accuracy for sounds presented in the back space, when pointing with the hand [*t*_(10)_ = 5.5, *P* = 0.001] and a trend of the same pattern emerged when pointing with the foot [*t*_(10)_ = 2.5, *P* = 0.1, *P* uncorrected (0.03)]. This suggests, therefore, that the effector was not the main cause of the bias.

To determine the contribution of *x*- and *y*-axes in the localization error, we performed an analysis on bias.

### Analysis on Bias

**FIGURE 4:**
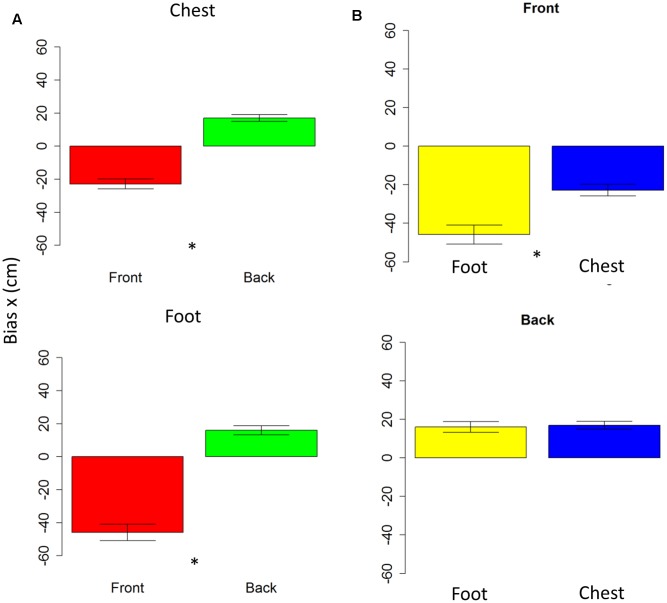
Bias in motor pointing task. **(A)** Left plots compares front (red bar) and back (green bar) bias for sounds presented at high level (upper plot) and low level (lower plot). As can be seen a greater bias is present in the frontal space than in the back. **(B)** Right plots compare low (yellow bar) and high (blue bar) sound level, in the frontal (upper plot) and back (lower plot) spaces. As can be seen, there is a significant difference between sound elevations in the frontal space, while no difference in the back space. Significant differences are illustrated (^∗^*P* < 0.05).

Anova on bias showed no differences on the *y*-axis (all *P* > 0.05); while on the *x*-axis, spatial bias emerged. The bias is specific for the frontal space [*F*_(1,10)_ = 5.7, *P* < 0.001], showing that subjects perceived frontal sound toward the back. The bias is present at foot level [*t*_(10)_ = -0.7, *P* < 0.001] and at chest level [*t*_(10)_ = 7.6, *P* < 0.001], as shown in **Figure 4A**. Interestingly, when comparing rear space at foot and chest level, no bias is reported [*t*_(10)_ = 0.38, *P* = 1], while when comparing chest and foot frontal spaces, a strong bias appears at foot level [*t*_(10)_ = -4, *P* = 0.005], as can be seen in **Figure 4B**. Bias on longitudinal space is not affected by the effector adopted [*F*_(1,10)_ = 3.6, *P* = 0.01]. However, effector influences bias on sound elevation [*F*_(1,10)_ = 2.22, *P* = 0.6], showing similar results with hand and foot for sound delivered at chest level [*t*_(10)_ = -0.23, *P* = 1] and a smaller bias at foot level, when pointing was performed with the foot [*t*_(10)_ = -32, *P* = 0.02]. These data shows that localization error was mainly due to a bias on the *x*-axis and not on the *y*-axis.

### Beta Regression Model

**FIGURE 5:**
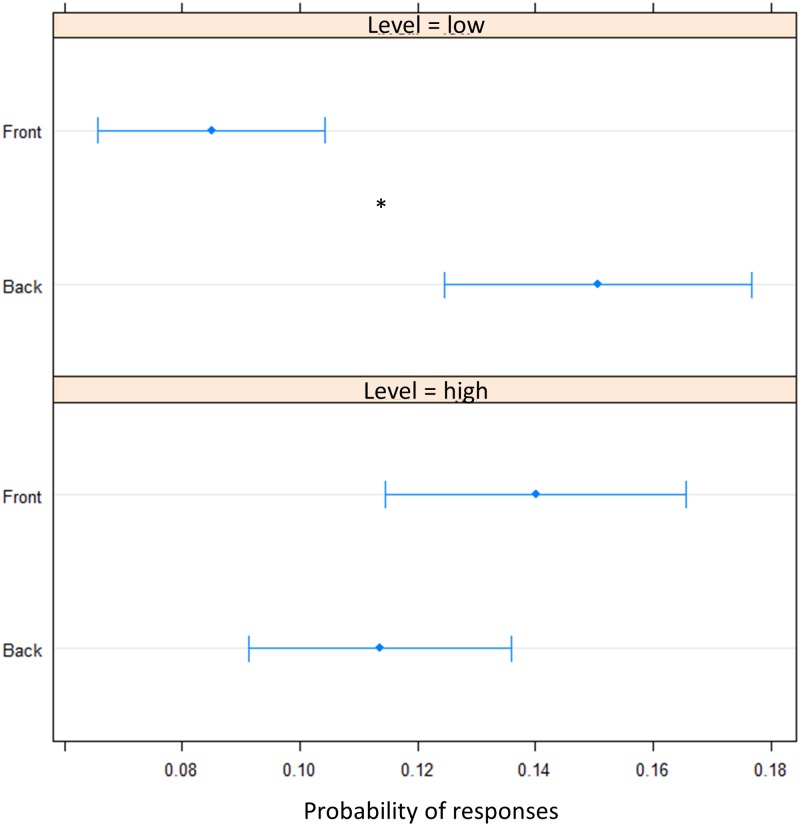
Proportion of verbal of answers in motor pointing task: the plot represents the amount of front and back answers given for sounds presented at low level and at high level. As can be seen a greater number of ‘back’ responses is present at low level, while at high level, no difference is present between the two longitudinal space. Significant differences are illustrated (^∗^*P* < 0.05).

To elucidate whether the bias was due to an overturning (i.e., sound presented in the front perceived in the back) perception or to a shift toward the back of pointing (i.e., perceived as closer to the body but still in the same hemifield), we fitted a beta regression model for proportions of responses. In the motor pointing, analysis on frequencies once again showed an influence of sound elevation (chest vs. foot level) on the longitudinal plane (front vs. back) [*X*^2^_(1)_ = 18.40, *P* < 0.001]. There was no differences between “Front” and “Back” responses for sound delivered at chest level [(OR) = -0.02 ± 0.01, *z*.ratio = -1.52, *P* = 0.11], while a greater number of “Back” responses were given at foot level [(OR) = 0.06 ± 0.01, *z*.ratio = 4.09, *P* < 0.001] (**Figure 5**). Interestingly, no differences between high and low spaces were found in the back [(OR) = -0.03 ± 0.01, *z*.ratio = -2.161, *P* = 0.03], while in the frontal space a lower number of ‘frontal’ answers were given at foot level [(OR) = 0.05 ± 0,01, *z*.ratio = 3.48, *P* = 0.0005]. Moreover, independently from elevation, transversal position (left vs. right) influences sound localization on longitudinal position (front vs. back), showing a greater number of “back” responses for sound presented on the left [(OR) = 0.09, *z*.ratio = 4.87, *P* < 0.001], while a greater amount of front answer for sound delivered on the right [(OR) = -0.05, *z*.ratio = -3.74, *P* = 0.0002].

**FIGURE 6:**
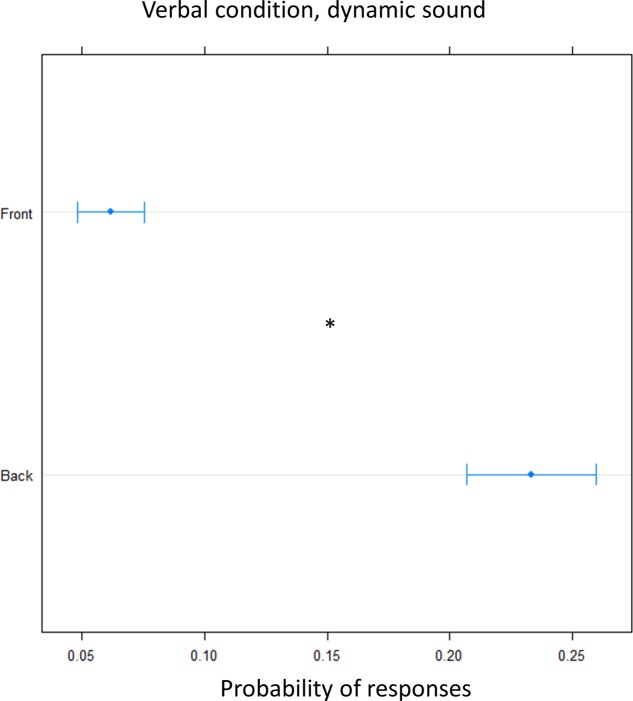
Proportion of verbal answers in the verbal pointing task with dynamic sound: the plot represents the number of front and back answers given, independently of sound level. As can be seen a greater number of ‘back’ responses was reported. Significant differences are illustrated (^∗^*P* < 0.05).

In order to clarify the role of the motor response on the bias, we performed the task, in another group of subjects, asking them to give a verbal, instead of motor, response (verbal condition). A greater number of ‘back’ responses was given compared to frontal position [(OR) = 0.17 ± 0.01, *z*.ratio = 11.8, *P* = <0.0001]. This suggest that front back error toward the back was still present for sound delivered at foot level and it is now also present for sound delivered at chest level, leading to an overturning of the localization toward the back (**Figure 6**).

**FIGURE 7:**
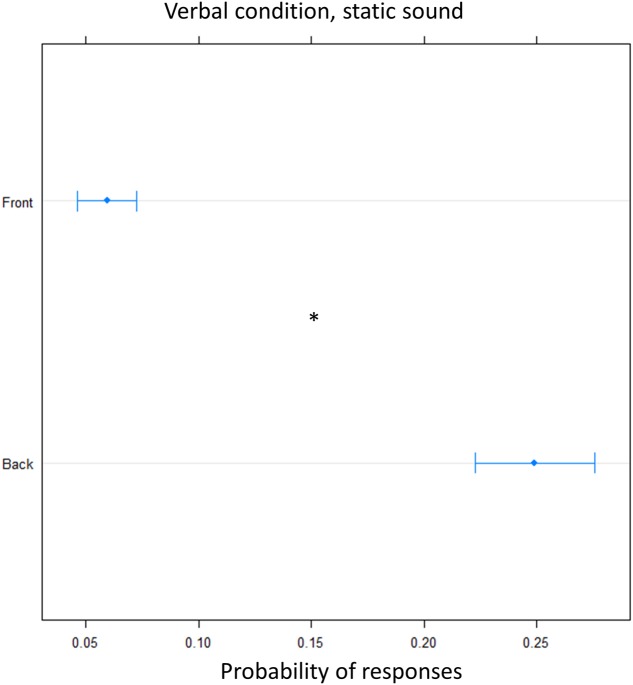
Proportion of verbal answers in the verbal pointing task with static sound: the plot represents the number of front and back answers given, independently of sound level. As can be seen greater number of ‘back’ responses were reported. Significant differences are illustrated (^∗^*P* < 0.05).

Finally, we tested whether the effect was specific to the dynamic audio stimulus used. To this aim the verbal condition was replicated using a static sound. Again, there was an overall higher frequency of ‘back’ answers [(OR) = 0.18 ± 0.01, *z*.ratio = 13, *P* < 0.001], showing that there was significant overturning of the localization toward the back, at both elevation (**Figure 7**).

## Discussion

In this study, we investigated the auditory space perception of different spaces around our body by analyzing the localization error of sound delivered at chest level and at foot level, both in the frontal and in the rear space. Furthermore, we evaluated the role of motor action in localizing static and dynamic sounds. Previous works showed that people are highly accurate in localizing frontal sounds delivered at head level. However, when the task requires location of sound delivered in the front or in the back, frequent reversal errors appear. As seen in the introduction, FB and BF errors are rare for open ear conditions and are most frequent for sound sources located on or near the median plane, (Makous and Middlebrooks, 1990). In order to minimize these biasing factors, we performed the task in real 3D space, with sounds slightly lateralized on the right and on the left. Our results showed that around the chest and around the feet, subjects are generally more accurate in localizing sounds presented in the back space. Contrarily, in the frontal zone they are less accurate and a large number of front–back errors are reported. This discrepancy between reversal errors had already been documented in literature (Chasin and Chong, 1999). Bias analysis revealed that localization error was mainly due to longitudinal space (*x*-axis), showing a great bias toward the back on both elevation. However, bias is not able to distinguish between reversal error (overturning) and shift toward the back. Beta regression model further clarified this point, by showing that at foot level a greater amount of front to back errors were present, while at chest level the bias was better explained by a shift toward the back. Interesting in the verbal condition, front–back errors were present at both elevation. Crucially, this pattern of data shows that movement can improve, by reducing front back error, sound localization in the space where we usually operate (i.e., high frontal space). Moreover, the fact that in all tasks the reversal error was unidirectional (from front to back) excludes the possibility that the effect was due to the cone of confusion. In general, we think that the greater localization error toward the back could be considered as an adaptive mechanisms due to the availability of different senses in that space. The visual modality is, indeed, crucial for space representation, but it is not available in the back space, where the auditory modality could be more salient (Thinus-Blanc and Gaunet, 1997; Gori et al., 2014; King, 2015; Cappagli et al., 2017). Studies on blind children and adults confirm the role of vision for spatial construction. Blind and low vision children and adults, for example, show specific deficits in some forms of audio space perception in front of them (Gori et al., 2014; Vercillo et al., 2016; Cappagli et al., 2017). For humans and most animals, vision is confined to frontal space, and mostly at head level (Oldfield and Parker, 1984; Kóbor et al., 2006). In order to look in the back space, humans have to turn their heads or their whole bodies. It is plausible, therefore that our brain develops different spatial representations based on the most reliable perceptual sense available in different spaces (i.e., vision in the front, hearing in the back). In agreement with this idea, it has been shown that, when audio–visual stimuli are delivered frontally, vision dominates the final perception, weighting more in multisensory estimation (Alais and Burr, 2004). Our result are in agreement with this idea, as well as with studies on blind people, where subjects displayed better accuracy in the back space, compared to sighted people (Voss et al., 2004). Neuroimaging studies on blind and sighted people have shown that the visual cortex is specifically recruited to process subtle monaural cues more effectively (Gougoux et al., 2005; Voss et al., 2006). Similarly, in sighted people, it has been reported that monaural cues are more useful in differentiating between front and back sound source (Steinhauser, 1879; Batteau, 1967; Gardner and Gardner, 1973; Musicant and Butler, 1984; Lopez-Poveda and Meddis, 1996). Moreover, Gougoux et al. (2005) found that inhibitory patterns differ between early-blind and sighted individuals. He found that, during monaural sound localization (one ear plugged), the subgroup of early-blind subjects who were behaviourally superior at sound localization displayed two activation foci in the occipital cortex. This effect was not seen in blind and sighted people who did not have superior monaural sound localizations. The degree of activation of one of these foci was strongly correlated with sound localization accuracy across the entire group of blind subjects, showing that the blind people who perform better than sighted individuals recruit occipital areas to carry out auditory localization under monaural conditions. It can therefore be concluded that computations carried out in the occipital cortex specifically underlie the enhanced capacity to use monaural cues. This differential pattern may provide evidences as to how different parts of the brain normally interact during unimodal stimulation, and further suggests that these interactions may be modified in the absence of a sensory modality. A possible speculation based on our result could be that, as in blind individuals, sighted individuals could recruit the occipital cortex when they have to localize rear sound, as they never received visual information from the back. If this were the case, it would explain why they are better at localizing rear sound and are more prone to front back error, when sound are presented in the front; further neurophysiological studies will be performed to investigate this point. Our results are also in agreement with previous studies that showed different saliency of auditory stimuli in the rear space (Farnè and Làdavas, 2002; Zampini et al., 2007). Importantly, both these explanations are related to the simple localization task, while vision is necessary to develop a more refined spatial map, for example, that required in the spatial bisection task (Gori et al., 2014), Interestingly, the auditory localization of frontal and rear auditory space seems to be related, not only to the body part considered but also to the involvement of body movement. Indeed, in the motor condition, when subjects can perform actions as response, there is strong bias toward the back at both elevations, while greater front–back error is present only at foot level, but not at chest level.

In everyday activities, we operate mainly in the higher frontal space, where different perceptual stimuli (auditory and visual) are closely integrated and linked to motor action (i.e., grasping, pointing). The lower space could be considered as a special space, where actions are mediated by foot and audio and motor feedback are naturally linked during walking. The result obtained in this work suggests a different role of the sensory feedback and motor control, available in the two body spaces, leading to a more accurate representation of auditory frontal space around the chest, than of the frontal auditory representation around the foot. In addition, our data suggest that movement improves sound localization, reducing front back error.

In order to clarify the role of movement in localizing sounds, subjects were required to give a verbal answer regarding the end position of the sound. If the front back error was due to the effector used or to movement in general, it should have disappeared in the verbal condition. This was not the case at foot level, where there was still a great number of front to back errors were still present, suggesting that localization error at this level was due to different sensory representations of these two spaces. However, in the pointing motor task, the bias toward the back was present at both elevations, but only data at foot level were explained by front to back error, suggesting that motor command plays a significant role in discriminating front from back sounds. Crucially, in the verbal condition, front–back errors were also found at chest level. We think that the possibility to move significantly reduces error in localizing frontal sounds around the chest, probably because in this space we are used to integrating sensory feedbacks with actions (Goodale, 2011); so localizing sounds at this level could be seen as a sort of reaching (Perris and Clifton, 1988). Finally, our data show that sound features do not influence sound localization. Indeed, the same effect was observed with static and moving sounds. It is important to note that our task required discrimination between front and back and it was not a simple localization in one hemifield (i.e., left vs. right). We think that people are very good at localizing frontal auditory stimuli when the task involves only this space, as shown by a previous study (Gori et al., 2014) showing that sighted subjects were good in representing frontal auditory space. However, disambiguating the front from the back is difficult, as the hearing system has few tools to localize stimuli. However, as discussed above, we think that the fact that vision is unavailable in the back could make hearing more salient in this space so when our brain is uncertain on where the sound come from, it tends to locate it in the back space.

Our results suggest that different mechanisms are implied in representing different spaces around our body. In particular, we showed that movement influences the audio–visual representation of high frontal space. Increased accuracy, found in the motor condition for sounds delivered at chest level, is not related to a mere perceptual effect, otherwise we would have found higher accuracy over all spaces (front vs. back) around chest. Our results clearly indicates that front and back spaces are differently affected by sound elevation, as no difference was present in the back space, while in the frontal area it was possible to note more accurate performance for sound delivered at chest level, probably due to motor action. This pattern of accuracy could be due to different representations of frontal and rear auditory space, that in turn are mediated by a motor action.

## Conclusion

Our data showed a tendency to report frontal sound as coming from the back. This could be due to a shift toward the back or to a front to back error. Front to back errors explain the data found at foot level and in the oral task, while only a shift toward the back is present at chest level, when a motor pointing is required, suggesting that movement helps in discriminating front from back. We speculate that our brain build a representation of the space based on the reliability of sensory stimuli in those spaces. This could explain the greater number of front to back errors, suggesting that, when stimuli are not visible and auditory information is useless, back space becomes more salient, because there hearing is the only sense available to detect stimuli. This pattern could be due to adaptive mechanisms.

## Author Contributions

Contributions to the conception: EA-V, CC, SF, and MG. Data acquisition: EA-V, CC, and SF. Data analysis: EA-V, CC, and SF. Interpretation of data: EA-V, CC, SF, and MG. Drafting the work: EA-V. Revising it: EA-V, CC, SF, and MG. Final approval of the version to be published: EA-V, CC, SF, and MG.

## Conflict of Interest Statement

The authors declare that the research was conducted in the absence of any commercial or financial relationships that could be construed as a potential conflict of interest.
